# Screw Worms in the Nose

**Published:** 1891-12

**Authors:** T. J. Turpin

**Affiliations:** Laredo


### SCREW WORMS.

### REPLY TO DR. GILBERT'S REQUEST.

*Editor Daniel's Medical Journal:*

I notice in the November issue of your journal that Dr. Gilbert, of Arizona, asks "The best treatment for screw worms in the nose." This is quite a common occurrence here in man as as well as beast. Every doctor in Laredo has had one or more cases, I think.

I have had two cases lately, and found that the inhalation of a little chloroform causes the worms to drop out long before it causes insensibility in the patient. The after treatment consisted in keeping the nostril free from pus by the injection of Proxide of Hydrogen.

I have also used calomel, as suggested by Dr. Schmidt, of Schulenberg, Texas, but the chloroform is much the most effectual.

